# Heart failure with insulin degludec versus glargine U100 in patients with type 2 diabetes at high risk of cardiovascular disease: DEVOTE 14

**DOI:** 10.1186/s12933-019-0960-8

**Published:** 2019-11-15

**Authors:** Richard E. Pratley, Mansoor Husain, Ildiko Lingvay, Thomas R. Pieber, Thomas Mark, Hans A. Saevereid, Daniel Vega Møller, Bernard Zinman, the DEVOTE Study Group

**Affiliations:** 1 AdventHealth Translational Research Institute for Metabolism and Diabetes, 301 E. Princeton Street, Orlando, FL 32804 USA; 2 Peter Munk Cardiac Centre, University Health Network, Toronto, Canada; 3 Department of Medicine and the Heart and Stroke Richard Lewar Centre, University of Toronto, Toronto, Canada; 4 Toronto General Hospital Research Institute, University Health Network, Toronto, Canada; 5 Ted Rogers Centre for Heart Research, Toronto, Canada; 6 Department of Internal Medicine and Department of Population and Data Sciences, University of Texas Southwestern, Dallas, TX USA; 7 Department of Internal Medicine, Medical University of Graz, Graz, Austria; 8 Novo Nordisk A/S, Søborg, Denmark; 9 Lunenfeld–Tanenbaum Research Institute, Mt. Sinai Hospital, University of Toronto, Toronto, ON Canada

**Keywords:** Clinical trial, Hospitalization for heart failure, Severe hypoglycemia, Insulin degludec, Type 2 diabetes

## Abstract

**Background:**

Heart failure (HF) is a common cardiovascular complication of type 2 diabetes (T2D). This secondary analysis investigated baseline factors and treatment differences associated with risk of hospitalization for HF (hHF), and the possible association between severe hypoglycemia and hHF.

**Methods:**

DEVOTE was a treat-to-target, double-blind cardiovascular outcomes trial in patients (n = 7637) with T2D and high cardiovascular risk randomized to insulin degludec (degludec) or insulin glargine 100 units/mL (glargine U100). The main endpoint of this secondary analysis was time to first hHF (standardized MedDRA Query definition). Severe hypoglycemia was adjudicated (American Diabetes Association definition). The main endpoint and the temporal association between severe hypoglycemia and hHF were analyzed with a Cox proportional hazards regression model. Predictors of time to first hHF were identified using baseline variables.

**Results:**

Overall, 372 (4.9%) patients experienced hHF (550 events). There was no significant difference in the risk of hHF between treatments (hazard ratio [HR] 0.88 [0.72;1.08]_95% CI_, *p *= 0.227). Prior HF (HR 4.89 [3.90;6.14]_95% CI_, *p *≤ 0.0001) was the strongest predictor of future hHF events. The risk of hHF significantly increased after (HR 2.2), and within a week after (HR 11.1), experiencing a severe hypoglycemic episode compared with before an episode.

**Conclusions:**

In patients with T2D and high cardiovascular risk there were no treatment differences in terms of hHF. Prior HF was the strongest predictor of future hHF events, and there was an association between severe hypoglycemia and subsequent hHF. Further research should evaluate whether the risk of hHF can be modified by treatments aimed at reducing hypoglycemia.

*Trial Registration* NCT01959529

## Background

Type 2 diabetes (T2D) is becoming increasingly prevalent worldwide and is one of the leading causes of death in the United States [1, 2]. Patients with diabetes are two to three times more likely to have cardiovascular disease (CVD) compared with those without diabetes [3], and CVD is the leading cause of death among these patients [4]. The increased risk of CVD in patients with T2D may be mediated in part through sub-optimal glycemic control, especially chronic hyperglycemia [5, 6], but also hypoglycemia. With respect to the latter, a clear association has been established, although a direct causal relationship remains unclear [7–12]. As T2D progresses, maintaining glycemic control becomes more challenging, and many patients require treatment intensification using insulin. Insulin-treated T2D increases the risk of severe hypoglycemia [13], which is associated with an increased rate of cardiovascular (CV) events [14, 15] and the possible risk of hospitalization for heart failure (HF) [16–22].

T2D is an independent risk factor for the development of HF [16]. Indeed, HF has emerged as one of the most common CV complications of T2D [23, 24]. It is estimated that 6.5 million Americans have HF [25], and of these, approximately 40% are reported to have T2D [26]. HF is associated with a poor prognosis; 30–40% of those diagnosed with HF die within 1 year and from then on, mortality rates remain high at 9% per year [27]. Not surprisingly, patients with HF and diabetes have an increased risk of mortality compared with patients with diabetes and no HF [28]. With the prevalence of T2D and HF predicted to increase in the foreseeable future, the coexistence of HF and T2D will become even more pronounced [25].

Recognizing the importance of HF as a complication of T2D, it has been suggested to include HF in a broader 5-point major adverse CV event (MACE) definition when performing T2D CV outcomes trials (CVOTs) [29–31]. A recent feasibility study using the Medical Dictionary for Regulatory Activities (MedDRA)-based endpoints suggested that these MedDRA query-derived endpoints may have utility as they closely approximate the adjudicated estimates reported in CVOTs [32].

Insulin degludec (degludec) is a basal insulin with an ultra-long duration of action, which has been shown to lead to lower rates of overall confirmed, nocturnal confirmed and severe hypoglycemia compared with insulin glargine 100 units/mL (glargine U100) across different patient populations, including the general diabetes population, elderly patients and patients with type 1 diabetes [33–40]. In the CVOT Trial Comparing Cardiovascular Safety of Insulin Degludec versus Insulin Glargine in Patients with Type 2 Diabetes at High Risk of Cardiovascular Events (DEVOTE), the CV safety of degludec versus glargine U100 was assessed in patients with T2D at high risk of CV events [38]. The primary analysis of DEVOTE showed that degludec was non-inferior to glargine U100 in terms of a 3-point MACE composite endpoint (CV death, non-fatal myocardial infarction [MI] and non-fatal stroke) (hazard ratio [HR] 0.91 [0.78; 1.06]_95% CI_) [38]. A 4-point MACE composite endpoint that included hospitalization for unstable angina demonstrated similar results. In addition, treatment with degludec, compared with glargine U100, resulted in a lower rate of both severe and nocturnal severe hypoglycemia (HR 0.60 [0.48; 0.76]_95% CI_ and 0.47 [0.31; 0.73]_95% CI_, respectively; both *p *< 0.001). In a pre-specified secondary analysis of DEVOTE, a temporal association between severe hypoglycemia and all-cause mortality was identified [11].

DEVOTE presents an opportunity to investigate the risk of HF in a large patient cohort (n = 7637) with T2D at high risk of CV events and treated with insulin. The aims of this pre-specified secondary analysis were to investigate baseline factors and treatment differences that are associated with an increased risk of hospitalization for HF (hHF), and to gain a better understanding of the possible association between severe hypoglycemia and the subsequent increased risk of hHF.

## Methods

### Trial design

The present pre-specified secondary analysis included patients from DEVOTE, a prospective, treat-to-target, randomized, double-blind, active-comparator CVOT. The trial was event driven, and designed to continue until at least 633 episodes of first MACE had occurred. The events (MACE and severe hypoglycemia, but not hHF) were confirmed by a central, blinded, independent Event Adjudication Committee (EAC). The median observation time was 2.0 years in both treatment arms. A more detailed description of the trial protocol, methods and the primary results has been published previously [38, 41].

DEVOTE is registered with ClinicalTrials.gov number NCT01959529 and was conducted in accordance with the Declaration of Helsinki and ICH Good Clinical Practice Guideline [42, 43]. The protocol was approved by independent ethics committees or institutional review boards for each center; written informed consent was obtained from each patient before any trial-related activities.

### Patients and treatments

Eligible patients included those with T2D treated with at least one oral or injectable antihyperglycemic agent with A1C ≥ 7.0% (53 mmol/mol), or with ≥ 20 units/day of basal insulin, and were either aged ≥ 50 years with at least one co-existing CV or renal condition, or aged ≥ 60 years and had at least one pre-specified CV risk factor.

Patients were randomized 1:1 to receive either degludec or glargine U100 administered once daily between dinner and bedtime, in addition to standard of care. As the study was double-blinded, both treatments were provided in identical 100 U/mL, 10 mL vials. All patients were allowed to continue their pre-trial antihyperglycemic therapy with the exception of basal and premix insulin, which were discontinued.

### Endpoints

The primary endpoint in DEVOTE was the time from randomization to first occurrence of MACE, a composite of death from CV causes, non-fatal MI or non-fatal stroke. The main endpoint of this secondary analysis was time to first hHF, an endpoint not adjudicated by the EAC. This endpoint was defined using the standardized MedDRA Query (SMQ; version 19.0) definition of cardiac failure which is restricted to specific terminology and symptoms, signs and investigational findings that are pathognomonic for cardiac failure (see Additional file 1: Additional Methods) along with a requirement for hospitalization, defined as an admission to an inpatient unit or a visit to an emergency department requiring at least a 12-h stay. The number of EAC-confirmed severe hypoglycemic episodes was a confirmatory endpoint. Severe hypoglycemia was defined in accordance with the American Diabetes Association criteria as an episode requiring the assistance of another person to actively administer carbohydrate or glucagon or to take other corrective actions [44].

### Statistical analysis

The main endpoint and associated sensitivity analyses were analyzed with a Cox proportional hazards regression model, with treatment group as a fixed factor. To identify significant predictors of time to first hHF, available baseline and medical history variables were considered in a stepwise model selection procedure in SAS PHREG [45] with p-value thresholds of 0.1 and 0.05 determining whether a single predictor should be added or removed from the model, respectively. Based on a Cox regression model that included all the significant baseline predictors simultaneously, the relative importance of a baseline predictor of hHF was calculated based on the Chi square contribution of each variable relative to the total Chi square. This indicated the relative degree to which baseline variables could predict hHF.

Several different sensitivity analyses were conducted. The time to first hHF was also defined by a broad MedDRA search that included patients with signs, symptoms or investigational findings highly suggestive of cardiac failure; this search also included the requirement for hospitalization. In another, a pre-specified set of baseline variables (sex, region, age, estimated glomerular filtration rate [eGFR], smoking status, diabetes duration, CV risk, and whether or not the patients were insulin naïve) were included as explanatory effects in the model. Similar sensitivity analyses were carried out for the primary endpoint in DEVOTE, as reported previously [38]. An additional sensitivity analysis was conducted that analyzed time to first hHF (SMQ definition) or HF leading to death (SMQ definition).

As hHF was not adjudicated in DEVOTE, two additional sensitivity analyses were conducted using information from the Liraglutide Effect and Action in Diabetes: Evaluation of Cardiovascular Outcome Results (LEADER) trial adjudication of hHF (that utilized the broad MedDRA search), which included a patient population at high risk of CV events similar to DEVOTE [46]. Based on LEADER data, the proportion of positively adjudicated hHF relative to all adjudicated hHF events was calculated. These LEADER positive adjudication probabilities were used to resample the data from DEVOTE for a weighted analysis (see Additional file 1: Table S1). Serious adverse events captured by the broad MedDRA search were picked at random with a probability equal to the LEADER adjudication probabilities. This random resampling was repeated 100 times and mean values were reported. For the few preferred terms available in DEVOTE which were not available in LEADER, probabilities of 100% and 0%, respectively, were used in two separate sensitivity analyses. It should be noted that the preferred terms used in the adjudication of hHF were more extensive than those used in the DEVOTE SMQ definition.

In the main analysis, missing data were assumed to be missing at random. In order to assess the robustness of this assumption with respect to the conclusions, a tipping point analysis was conducted. In this case, non-informative censored patients randomized to degludec were assumed to have hHF the day after being censored, starting with the earliest non-informative censored patient relative to the individual randomization date and then moving forward until the upper-bound of the 95% confidence interval (CI) was above the pre-specified non-inferiority limit of 1.3 or until end.

Severe hypoglycemia and subsequent risk of experiencing hHF were analyzed with a Cox proportional hazards regression model with treatment and previous experience of severe hypoglycemia (Yes/No) as a time-varying covariate. Similar analyses to adjust for general frailty were carried out using the following baseline variables: sex, age, smoker status, geographic region from (US/Non-US), diabetes duration, insulin naïve, CV risk as fixed factors as well as eGFR and A1C as fixed covariates.

## Results

### hHF and baseline characteristics

Using the SMQ definition of hHF, 372 (4.9%) patients experienced hHF, reporting a total of 550 events during the 2 years of observation. Of these events, 499 were classed as cardiac disorders (see Additional file 1: Table S2). Of those patients who experienced hHF during the trial, 58.9% had a diagnosis of HF before the trial. Using the broad MedDRA search with the LEADER match, there were 618 (8.1%) patients who experienced hHF, with 948 reported events, the majority (784 events) of which were classed as cardiac disorders (see Additional file 1: Table S2).

Several baseline characteristics were significantly different for patients who experienced hHF during the trial compared with those who did not (Table 1). Overall, 93.8% of patients who experienced hHF during the trial had established CVD/chronic kidney disease (CKD) at baseline, compared with 84.8% of patients not experiencing hHF (*p *< 0.0001). Patients who experienced hHF during the trial had a higher body mass index versus those who did not (35.5 kg/m^2^ vs. 33.5 kg/m^2^, *p *< 0.001) and a lower eGFR (59.0 mL/min/1.73 m^2^ vs. 68.4 mL/min/1.73 m^2^, *p *< 0.001). Additionally, a higher proportion of patients who experienced hHF during the trial had hepatic impairment (9.9%) compared with those who did not have hHF (2.2%) (*p *< 0.0001).

**Table 1:** Baseline characteristics and medical history by hHF during the trial

	hHF during trial, n = 372	No hHF during trial, n = 7265	p-value
Age, years	65.8 (± 8.1)	64.9 (± 7.3)	0.022
Male, n (%)	222 (59.7)	4556 (62.7)	NS
Region, n (% from North America)	299 (80.4)	4972 (68.4)	0.0001
Established CVD/CKD ≥ 50 years, n (%)	349 (93.8)	6160 (84.8)	< 0.0001
Hepatic impairment, n (%)	37 (9.9)	159 (2.2)	< 0.0001
Current smoker, n (%)	40 (10.8)	812 (11.2)	NS
Insulin naïve, n (%)	36 (9.7)	1192 (16.4)	0.0006
A1C, %	8.6 (± 1.8)	8.4 (± 1.6)	NS
FPG, mmol/L	9.8 (± 4.5)	9.5 (± 3.9)	NS
Duration of diabetes, years	17.6 (± 9.1)	16.4 (± 8.9)	0.006
BMI, kg/m^2^	35.5 (± 7.3)	33.5 (± 6.8)	< 0.001
Body weight, kg	101.2 (± 23.8)	95.8 (± 22.8)	< 0.001
eGFR, mL/min/1.73 m^2^	59.0 (± 22.6)	68.4 (± 21.4)	< 0.001
Systolic blood pressure (mmHg)	137.1 (± 21.2)	135.5 (± 17.9)	NS
Diastolic blood pressure (mmHg)	75.1 (± 12.4)	76.2 (± 10.3)	0.039
Pulse, beats/min	73.2 (11.6)	73.1 (11.3)	–
Prior heart failure, n (%)	219 (58.9)	1115 (15.3)	–
Prior myocardial infarction, n (%)	182 (48.9)	2424 (33.4)	–
Atrial fibrillation, n (%)	97 (26.1)	627 (8.6)	–
Macular edema, n (%)	5 (1.3)	24 (0.3)	–
Proteinuria (microalbuminuria and gross proteinuria), n (%)	107 (28.8)	1710 (23.5)	–
Insulin, n (%)			
Long acting	241 (64.8)	4356 (60.0)^b^	–
Intermediate acting^a^	59 (15.9)	1015 (14.0)^b^	–
Bolus	186 (50.0)	2645 (36.4)^b^	–
Premix	46 (12.4)	736 (10.1)^b^	–
Antihypertensive therapy, n (%)			
Beta-blockers	279 (75.0)	4121 (56.7)^c^	–
Calcium channel blockers	136 (36.6)	2322 (32.0)^c^	–
Angiotensin-converting enzyme inhibitors	163 (43.8)	3464 (47.7)^c^	–
Angiotensin receptor blockers	139 (37.4)	2416 (33.3)^c^	–
Others	62 (16.7)	715 (9.8)^c^	–
Diuretics, n (%)			
Loop diuretics	226 (60.8)	1512 (20.8)^c^	–
Thiazides	68 (18.3)	1674 (23.0)^c^	–
Others	95 (25.5)	976 (13.4)^c^	–
Lipid-modifying medications, n (%)			
Statins	297 (79.8)	5705 (78.5)^c^	–
Fibrates	44 (11.8)	807 (11.1)^c^	–
Ezetimibe	18 (4.8)	328 (4.5)^c^	–
Others	11 (3.0)	257 (3.5)^c^	–
Platelet aggregation inhibitors, n (%)			
Acetylsalicylic acid	253 (68.0)	4739 (65.2)^c^	–
Others	108 (29.0)	1689 (23.2)^c^	–
Anti-thrombotic medication, n (%)	78 (21.0)	519 (7.1)^c^	–

Values are mean (± SD), unless otherwise stated

Hepatic impairment defined as having a score of > 2 on a modified Child–Pugh criteria scale using only bilirubin and albumin values

*A1C* glycosylated hemoglobin, *BMI* body mass index, *CKD* chronic kidney disease, *CVD* cardiovascular disease, *eGFR* estimated glomerular filtration rate, *FPG* fasting plasma glucose, *hHF* hospitalization for heart failure, *NS* not statistically significant, *SD* standard deviation

^a^Intermediate acting insulin cover human insulin, neutral protamine Hagedorn and unknown types of insulin

^b^Six patients have missing initiation drug date; they are assumed to be on treatment at baseline

^c^Nine patients have missing initiation drug date; they are assumed to be on treatment at baseline

In patients who experienced hHF during the trial versus those who did not experience hHF during the trial, a greater proportion at baseline used bolus insulin (50.0 vs. 36.4%), β-blockers (75.0 vs. 56.7%), diuretics (77.7 vs. 48.5%) and anti-thrombotics (21.0 vs. 7.1%), respectively.

Prior HF (HR 4.89), prior hepatic impairment (HR 3.08), eGFR (log regression) (HR 0.44), prior atrial fibrillation (HR 1.95), total insulin dose at week 1 (HR 1.53) and prior MI (HR 1.54) were all associated with a significantly higher risk of experiencing hHF during the trial (all *p *≤ 0.0001), with a relative importance (i.e. the relative degree to which baseline variables could predict hHF) of 54.6%, 11.0%, 10.0%, 7.2%, 5.9% and 4.3%, respectively. Other significant baseline predictors included macular edema, A1C, proteinuria and systolic blood pressure (Table 2). Baseline variables considered, but not having a significant effect on time to first hHF are listed in Additional file 1: Additional Methods.

**Table 2:** Predictors of time to first hHF (SMQ definition)

Baseline predictor	Hazard ratio [95% CI]	Relative importance	P-value
Prior heart failure (Y vs. N)	4.89 [3.90; 6.14]	54.6	< 0.0001
Hepatic impairment (Y vs. N)	3.08 [2.15; 4.41]	11.0	< 0.0001
eGFR (log regression)	0.44 [0.34; 0.58]	10.0	< 0.0001
Atrial fibrillation (Y vs. N)	1.95 [1.50; 2.55]	7.2	< 0.0001
Total insulin dose (U/kg) at week 1	1.53 [1.27; 1.84]	5.9	< 0.0001
Prior myocardial infarction (Y vs. N)	1.54 [1.23; 1.91]	4.3	0.0001
Macular edema (Y vs. N)	3.77 [1.40; 10.2]	2.0	0.0087
A1C (squared regression)	1.00 [1.00; 1.01]	1.8	0.0137
Proteinuria (microalbuminuria and gross proteinuria)	1.36 [1.06; 1.73]	1.8	0.0140
Systolic blood pressure at baseline	1.01 [1.00; 1.01]	1.5	0.0251

Variables identified by stepwise selection − FAS. Relative importance is calculated as 100 × Chi square/Total Chi square, where the Chi squares are from a model simultaneously considering all effects mentioned in the table

*A1C* glycosylated hemoglobin, *CI* confidence interval, *eGFR* estimated glomerular filtration rate, *FAS* full analysis set, *hHF* hospitalization for heart failure, *N* no, *SMQ* standardized Medical Dictionary for Regulatory Activities Query, *U* units, *Y* yes

### Treatment differences in time to first hHF

Using the SMQ definition, the main endpoint (hHF) occurred in 4.6% of patients with a rate of 3.42 events/100 patient-years of observation (PYO) in the degludec group and in 5.2% of patients with a rate of 3.85 events/100 PYO in the glargine U100 group (HR 0.88 [0.72; 1.08]_95% CI_, *p *= 0.227). A 5-point MACE composite endpoint (CV death, non-fatal MI, non-fatal stroke, hospitalization for unstable angina and hHF) demonstrated similar results to the primary 3-point MACE composite endpoint (HR 0.92 [0.82; 1.04]_95% CI_).

**Fig. 1:**
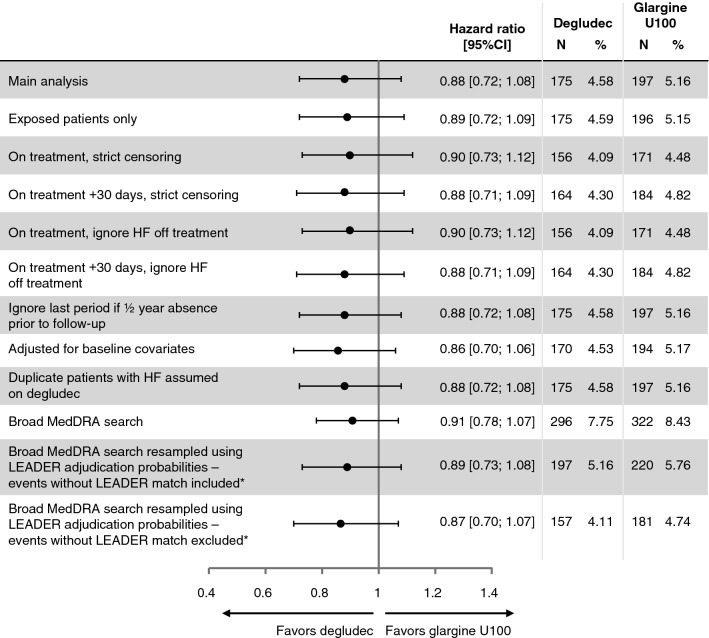
Main and sensitivity analyses of treatment differences in time to first hHF (SMQ definition). *Broad MedDRA search weighted by proportion confirmed in LEADER by the Event Adjudication Committee. There were only 100 events using the broad MedDRA search without the LEADER match, 31 of which were classed as cardiac disorders. *CI* confidence interval, *glargine U100* insulin glargine 100 units/mL, *hHF* hospitalization for heart failure, *HF* heart failure, *N* number of patients, *%* proportion of patients, *SMQ* standardized Medical Dictionary for Regulatory Activities Query

All sensitivity analyses (e.g. using the broad MedDRA search, exposed patients only, various censoring definitions, disregarding hHF off-treatment, adjusting for baseline covariates, and broad MedDRA resampled according to LEADER adjudication probabilities) were consistent with the findings of the main analysis, showing that the risk of hHF was not substantially different between degludec and glargine U100 (HR from 0.87 to 0.91, with all 95% CIs including 1.0) (Fig. 1). Using the broad MedDRA search, hHF occurred in 7.8% of patients with a rate of 5.80 events/100 PYO in the degludec group and in 8.4% of patients with a rate of 6.73 events/PYO in the glargine U100 group (HR 0.91 [0.78; 1.07]_95% CI_, *p *= 0.251). There were 12 patients (0.16%; degludec: n = 7; glargine U100: n = 5) who had HF leading to death but no hHF. When these events were included in the SMQ analysis a similar result to the main result was observed (HR 0.90 [0.73; 1.09]_95% CI_, *p *= 0.276).

There were 64 patients in the degludec group and 67 patients in the glargine U100 group with non-informative censoring. In the tipping point analysis, having the most conservative assumption about these patients prematurely discontinuing the trial, it was assumed that patients in the degludec group did have hHF the day after the end of trial and those randomized to glargine U100 did not have hHF. To exceed the non-inferiority limit of 1.3 for the upper-bound of the 95% CI, it was estimated that it would be necessary to impute 38 (59%) additional first events for patients with incomplete information (non-informative censoring) treated with degludec in order to overturn the conclusion from the main endpoint. That is, with 38 additional first events added to the degludec arm the HR (degludec versus glargine U100) was 1.07 [0.88; 1.30]_95% CI_.

In the degludec treatment group, four patients were lost to follow up, compared with one patient in the glargine U100 treatment group. Four additional first events were imputed in the degludec group and after that, there was still a similar risk of time to first hHF with degludec versus glargine U100 (HR 0.90 [0.74; 1.11]_95% CI_).

### Temporal association between severe hypoglycemia and the subsequent risk of hHF

The risk of hHF (at any time until the end of the trial) more than doubled (HR 2.2, *p *= 0.0002) after experiencing an episode of severe hypoglycemia compared with before an episode (Table 3). In addition, the risk of hHF increased more than tenfold (HR 11.1, *p *< 0.0001) within 7 days of experiencing an episode of severe hypoglycemia compared with before and more than 7 days after the episode. When adjusting for different sets of baseline variables the strength of this temporal association weakened, but there was still a significantly higher risk of hHF after experiencing severe hypoglycemia.

**Table 3:** Temporal association between severe hypoglycemia and subsequent risk of experiencing hHF in DEVOTE and LEADER

Time window (days after each severe hypoglycemic event)	DEVOTE (SMQ definition)								LEADER (SMQ definition)								LEADER (EAC confirmed)							
	Degludec		Glargine U100		Main analysis		Sensitivity analysis^a^		Liraglutide		Placebo		Main analysis		Sensitivity analysis^a^		Liraglutide		Placebo		Main analysis		Sensitivity analysis^a^	
	N	E	N	E	HR^b^	p-value	HR^b^	p-value	N	E	N	E	HR^b^	p-value	HR^b^	p-value	N	E	N	E	HR^b^	p-value	HR^b^	p-value
0–end trial	173	10	238	18	2.2	0.0002	1.7	0.0119	106	8	136	17	3.0	< 0.0001	2.3	< 0.0001	108	5	141	17	3.2	< 0.0001	2.4	0.0001
0–7	173	1	238	2	11.1	< 0.0001	9.8	< 0.0001	106	1	136	3	32.7	< 0.0001	23.1	< 0.0001	108	0	141	2	N/A	–	N/A	–
8–end trial	173	10	238	18	1.6	0.0358	1.3	0.2507	106	8	136	17	2.3	<0.0001	1.7	0.0109	108	5	141	17	3.3	< 0.0001	2.4	< 0.0001

Total number of patients experiencing severe hypoglycaemia: DEVOTE, degludec n = 187, glargine U100 n = 252 [38]; LEADER, liraglutide n = 114, placebo n = 153 [46]

For events occurring on the same day as a hypoglycemic event, 0.5 days were added to the day of the event

*CV* cardiovascular, *E* number of events, *EAC* Event Adjudication Committee; *eGFR* estimated glomerular filtration rate, *glargine U100* insulin glargine 100 units/mL, *hHF* hospitalization for heart failure, *HR* hazard ratio, *N* number of patients, *N/A* analysis did not converge, *SMQ* standardized Medical Dictionary for Regulatory Activities Query

^a^Adjusted for sex, region, age, eGFR, smoking status, diabetes duration, CV risk, and whether or not the patients were insulin naïve

^b^hHF with prior severe hypoglycemia in window versus hHF without severe hypoglycemia in window

When the SMQ definition of hHF was applied to LEADER, similar results were observed compared with the DEVOTE SMQ hHF analysis and the LEADER EAC-confirmed hHF analysis, even after adjusting for baseline variables (Table 3). Similarly in the LEADER trial, patients experiencing severe hypoglycemia were at significantly higher risk of subsequent EAC-confirmed hHF before the end of the trial than those who did not experience severe hypoglycemia (Table 3).

## Discussion

The results from this pre-specified secondary analysis of DEVOTE demonstrated that there was no significant difference in the risk of experiencing hHF with degludec versus glargine U100.

Prior HF at baseline was the strongest predictive factor for experiencing hHF during the trial. Other baseline factors associated with hHF during the trial included hepatic impairment, lower eGFR, atrial fibrillation, higher total insulin dose at week 1, prior MI, macular edema, higher A1C, proteinuria and higher systolic blood pressure. Furthermore, severe hypoglycemia during the trial increased the risk of subsequent hHF. These baseline characteristics are all hallmark characteristics of long-standing diabetes as well as both hepatic impairment and atrial fibrillation potentially being signs of established HF. In particular, a recent survey highlighted that patients with T2D with high blood glucose levels (> 11.1 mmol/L) admitted to hospital with HF had an increased mortality risk [47]. Furthermore, glycemic variability has also been shown to be independently related to an increased risk of all-cause mortality in patients with T2D and HF [48]. When simultaneously considering other baseline variables, diabetes duration was not a strong predictor of hHF. Likewise, age was significantly associated with hHF during the trial in a single-factor analysis, but when considering other predictors of hHF simultaneously this association was no longer significant. Therefore, suggesting that it is the complications associated with age and diabetes duration, and not the age or diabetes duration in itself, leading to an increased risk of hHF.

In this study, 58.9% of patients who experienced hHF had a diagnosis of HF before the trial, compared with 15.3% of patients who did not experience hHF. A greater proportion of patients who experienced hHF during the trial were using β-blockers and diuretics at baseline compared with those who did not experience hHF during the trial. This was expected, as these treatments are standard of care for patients with a prior diagnosis of HF.

Treatment with insulin increases the risk of severe hypoglycemia [13], which is associated with an increased risk of all-cause mortality and CV events, including stroke, coronary heart disease, CV disease and all-cause hospitalization (including heart failure) [16–22]. In a previous secondary analysis of DEVOTE, it was demonstrated that there was a significant association as well as a temporal association between severe hypoglycemia and all-cause mortality [11]. These results are similar to those observed in the LEADER trial, where it was demonstrated that patients experiencing severe hypoglycemia were more likely than those without severe hypoglycemia to experience MACE, CV death and all-cause mortality [8]. Analyses from the Veterans Affairs Diabetes Trial (VADT) also demonstrated that a severe hypoglycemic event was an independent predictor of death at 90 days [44, 49]. Furthermore, in the Action to Control Cardiovascular Risk in Diabetes (ACCORD) trial, patients who had one or more severe hypoglycemic episodes had higher rates of death than those who did not experience such episodes [50]. In the Outcome Reduction with Initial Glargine Intervention (ORIGIN) trial, severe hypoglycemia increased the risk of arrhythmic death, all-cause death and CV death [10]. In addition, in the Trial Evaluating Cardiovascular Outcomes with Sitagliptin (TECOS), severe hypoglycemia was associated with an increased risk of CV events, all-cause death and CV death [12]. In the current analysis, patients who experienced severe hypoglycemia were at a significantly higher risk of subsequently experiencing hHF than those who did not experience severe hypoglycemia. A strong temporal relationship (with the highest risk observed within a week of the event) further supports this hypothesis. Furthermore, such an association was also demonstrated in LEADER. The relatively weaker strength of an association when adjusting for baseline covariates suggests that the association may partly be explained by more frail patients suffering from comorbidities and complications, which in itself are risk factors of both severe hypoglycemia and hHF [12, 51]. Furthermore, although the hazard ratio for hHF did not reach statistical significance, the point estimate was in favor of degludec. This is consistent with the significantly lower rates of severe hypoglycemia with degludec versus glargine U100 observed in the primary DEVOTE trial [38, 41], and the significantly higher risk of hHF following an occurrence of severe hypoglycemia observed in this secondary analysis. The reason for the hazard ratio for hHF not reaching statistical significance, despite the other two strong significant associations, may be because factors other than treatment may affect the risk of severe hypoglycemia. Furthermore, the trial was not powered to detect a significant treatment effect for hHF. It is also important to note that our investigation into the possible association between severe hypoglycemia and subsequent hHF is relatively novel in that, aside from the TECOS and EMPA-REG OUTCOME trials, this association has not been explored by other trials [12, 52]. In TECOS, a significant association between severe hypoglycemia and subsequent hHF was not identified [12]. However, a post hoc exploratory analysis of the EMPA-REG OUTCOME trial demonstrated that hypoglycaemia was associated with an increased risk of subsequent hHF [52]. Our results are in line with those from EMPA-REG OUTCOME trial as well as LEADER, which had a similar patient population to DEVOTE. However, it is currently unclear whether there is a direct pathophysiological link between severe hypoglycemia and hHF, or whether severe hypoglycemia is primarily a marker of vulnerability for patients at risk of hHF. Overall, our results support an association between severe hypoglycemia and subsequent hHF even when adjusting for potential confounders and it is most likely that hypoglycemia is just a single contributory factor to hHF events in a much larger multifactorial landscape.

This study has a number of limitations. This was a pre-specified secondary analysis from a trial that was not powered to compare differences in the risk of hHF when treated with different basal insulin. It was also not designed or powered to compare the difference in risk of experiencing hHF based on HF prior to the trial or indeed the relationship between severe hypoglycemia and risk of hHF. However, the findings support those from other trials and therefore, this analysis supports and adds to the existing body of evidence.

Furthermore, the inclusion criteria of this trial were designed to recruit a cohort who were at a high CV risk, so the different outcomes observed may not be generalizable to the wider T2D population or to those not fulfilling the inclusion criteria. A further limitation is that hHF events were not adjudicated by an EAC in this trial. However, the sensitivity analysis based on LEADER adjudicated probabilities demonstrated similar results to analyses without adjudication.

Strengths of this study include the large sample size, the double-blind active-control design and the independent adjudication of severe hypoglycemic events. The prospective design and international multicenter nature of this trial, as well as the high levels of patient follow-up are additional strengths. Robustness was also increased through the use of a MedDRA search matched with LEADER EAC criteria, as well as use of the SMQ definition.

## Conclusions

This secondary analysis from DEVOTE of patients with T2D at high risk of CV events, treated with basal insulin (degludec or glargine U100) demonstrated no treatment differences with degludec versus glargine U100 in terms of hHF, that prior HF was the strongest predictor of future hHF events, and that there was an association between severe hypoglycemia and subsequent hHF, which was further supported by the similar results from LEADER.

## Supplementary information

**Additional file 1: Additional Methods.** Description of the SMQ definition of cardiac failure and the hHF definition used by the EAC in LEADER. **Table S1.** LEADER positive adjudication probabilities. **Table S2.** hHF by system organ class: hHF events by SMQ and broad MedDRA definitions.

## Data Availability

The datasets analyzed during the current study are available from the corresponding author on reasonable request.

## Abbreviations

A1C: glycated hemoglobin
ACCORD: Action to Control Cardiovascular Risk in Diabetes
CI: confidence interval
CKD: chronic kidney disease
CV: cardiovascular
CVD: cardiovascular disease
CVOTs: cardiovascular outcomes trials
Degludec: insulin degludec
DEVOTE: Trial Comparing Cardiovascular Safety of Insulin Degludec versus Insulin Glargine in Patients with Type 2 Diabetes at High Risk of Cardiovascular Events
EAC: Event Adjudication Committee
eGFR: estimated glomerular filtration rate
Glargine U100: insulin glargine 100 units/mL
hHF: hospitalization for HF
HF: heart failure
HR: hazard ratio
LEADER: Liraglutide Effect and Action in DiabetesEvaluation of Cardiovascular Outcome Results
MACE: major adverse cardiovascular events
MedDRA: Medical Dictionary for Regulatory Activities
MI: myocardial infarction
ORIGIN: Outcome Reduction with Initial Glargine Intervention
PYO: patient-years of observation
SMQ: standardized MedDRA Query
T2D: type 2 diabetes
TECOS: Trial Evaluating Cardiovascular Outcomes with Sitagliptin
VADT: Veterans Affairs Diabetes Trial
