# Supplementary material for: Heart failure with insulin degludec versus glargine U100 in patients with type 2 diabetes at high risk of cardiovascular disease: DEVOTE 14
Source: Cardiovasc Diabetol. 2019 Nov 15;18:156. doi: 10.1186/s12933-019-0960-8 (PMC6858747; doi:10.1186/s12933-019-0960-8)
Supplement: Supplementary file 1 — Additional file 1: Additional Methods. Description of the SMQ definition of cardiac failure and the hHF definition used by the EAC in LEADER. Table S1. LEADER positive adjudication probabilities. Table S2. hHF by system organ class: hHF events by SMQ and broad MedDRA definitions. [file 12933_2019_960_MOESM1_ESM.docx]

**ADDITIONAL FILE 1**

**Heart failure with insulin degludec versus glargine U100 in patients with type 2 diabetes at high risk of cardiovascular disease: DEVOTE 14**

**CONTENTS**

[Additional Methods 2](#_Toc15393600)

[Additional Table S1: LEADER positive adjudication probabilities 5](#_Toc15393601)

[Additional Table S2: hHF requiring hospitalization by system organ class 7](#_Toc15393602)

# Additional Methods

**SMQ definition of cardiac failure**

- Cardiac failure is defined as:
  - A condition in which the heart is unable to pump an adequate amount of blood to meet metabolic and physiological needs of body.
  - Classified on the basis of severity according to criteria set by New York Heart Association (NYHA) 0 Classes I to IV starting from no limitation of physical activity to slight or marked limitation, up to the inability to carry out any physical activity without discomfort.
  - Clinical findings vary but include: dependent edema, raised jugular venous pressure, hepatomegaly, pulmonary congestion/edema, tachycardia, cardiomegaly and dyspnea.
  - Cardiac ejection fraction is less than 35%.
- Inclusion/exclusion criteria (this SMQ does not make a distinction between left and right ventricular failure):
  - Included:
    - Narrow scope:
      - Terms describing existing cardiac failure in its various forms with or without right/left ventricular specified.
      - A small number of terms for symptoms, signs and investigational findings that are pathognomonic of the condition.
    - Broad scope:
      - Signs, symptoms or investigational findings highly suggestive of current or past evidence for this condition.
      - Cardiac cirrhosis.
  - Excluded:
    - Causality terms of cardiac failure, such as ventricular tachyarrhythmia or myocardial infarction.
    - Signs and symptoms that may result from cardiac failure, but are shared with so many other etiologies that they would merely serve to add noise or confusion to the search (e.g. dyspnea other than paroxysmal nocturnal dyspnea and orthopnea).
    - General effects of cardiac failure on the liver and kidney**.**

**hHF definition used by the EAC in LEADER**

- hHF is defined as an event that meets the following criteria:
  - Requires hospitalization defined as an admission to an inpatient unit or a visit to an emergency department that results in at least a 12-hour stay (or a date change if the time of admission/discharge is not available).

AND

- - Clinical manifestations of HF including at least one of the following: new or worsening dyspnea, orthopnea, paroxysmal nocturnal dyspnea, edema, pulmonary basilar crackles, jugular venous distension, new or worsening third heart sound or gallop rhythm, or radiological evidence of worsening heart failure.

AND

- - Additional/increased therapy, initiation of intravenous diuretic, inotrope or vasodilator therapy, up-titration of intravenous therapy, if already on therapy, initiation of mechanical or surgical intervention (mechanical circulatory support, heart transplantation or ventricular pacing to improve cardiac function), or the use of ultrafiltration, hemofiltration, or dialysis that is specifically directed at treatment of heart failure, or biomarker results (e.g. brain natriuretic peptide) consistent with congestive heart failure will be supportive of this diagnosis.

**Baseline variables considered, but not having a significant effect on time to first hHF**

| Treatment | Height |
| --- | --- |
| Country | Logarithm of height |
| Age | Height squared |
| Age group | Insulin naive |
| Logarithm of age | Insulin treatment at baseline |
| Age squared | Simplified insulin baseline groups |
| Statins | Total dose (units) at week 1 |
| Antidiabetic treatment at baseline | Basal dose (units) at week 1 |
| BMI | Basal dose (units/kg) at week |
| BMI at baseline ≥30kg/m^2^ flag | Baseline bolus insulin use |
| Total cholesterol | Only bolus insulin at baseline flag |
| HDL | Sex |
| Logarithm of HDL | Race group |
| HDL squared | Race is White flag |
| LDL | Ethnicity |
| LDL cholesterol groups | Geographic region |
| Logarithm of LDL | From USA flag |
| LDL squared | Smoker status |
| LDL/HDL cholesterol ratio | Patients qualified for the recommended glycemic titration target flag |
| Triglycerides | Pulse |
| Cardiovascular risk group | Prior stroke flag |
| Diabetes duration ≥15 years flag | Stroke |
| Diabetes duration | Myocardial infarction |
| Diastolic blood pressure | Peripheral artery disease |
| eGFR (CKD-EPI) | Foot ulcer |
| eGFR squared | Retinopathy |
| Renal failure severity (CKD-EPI) | Neuropathy |
| Simplified renal status groups (2 types of grouping) | Depression |
| A1C at baseline ≥8% flag | Angina |
| A1C | Left ventricular hypertrophy |
| Logarithm of A1C | Cataract |
| A1C squared | History of amputation |

BMI, body mass index; eGFR, estimated glomerular filtration rate; CKD-EPI, Chronic Kidney Disease Epidemiology Collaboration; HDL, high-density lipoprotein; hHF, hospitalization for HF; LDL, low-density lipoprotein; USA, United States of America

# Additional Table S1: LEADER positive adjudication probabilities

| **Preferred term** | **Number of events sent for EAC adjudication** | **Number of events confirmed by EAC adjudication** | **Percentage** |
| --- | --- | --- | --- |
| Total number of events | 1217 | 768 | 63.1 |
| Cardiac failure congestive | 371 | 273 | 73.6 |
| Cardiac failure | 330 | 233 | 70.6 |
| Cardiac failure chronic | 61 | 45 | 73.8 |
| Atrial fibrillation | 34 | 12 | 35.3 |
| Pulmonary edema | 29 | 15 | 51.7 |
| Cardiac failure acute | 22 | 16 | 72.7 |
| Left ventricular failure | 21 | 17 | 81.0 |
| Acute pulmonary edema | 20 | 14 | 70.0 |
| Dyspnea | 13 | 6 | 46.2 |
| Left ventricular dysfunction | 13 | 7 | 53.8 |
| Atrial flutter | 9 | 2 | 22.2 |
| Ventricular tachycardia | 9 | 0 | 0.0 |
| Acute left ventricular failure | 8 | 6 | 75.0 |
| Coronary artery disease | 7 | 1 | 14.3 |
| Ischemic cardiomyopathy | 7 | 5 | 71.4 |
| Cardiogenic shock | 6 | 3 | 50.0 |
| Cardiopulmonary failure | 6 | 3 | 50.0 |
| Fluid overload | 6 | 4 | 66.7 |
| Acute respiratory failure | 5 | 2 | 40.0 |
| Edema peripheral | 5 | 2 | 40.0 |
| Pneumonia | 5 | 4 | 80.0 |
| Arrhythmia | 4 | 2 | 50.0 |
| Atrioventricular block second degree | 4 | 1 | 25.0 |
| Chronic obstructive pulmonary disease | 4 | 3 | 75.0 |
| Myocardial ischemia | 4 | 1 | 25.0 |
| Respiratory failure | 4 | 1 | 25.0 |
| Aortic stenosis | 3 | 0 | 0.0 |
| Cardiomyopathy | 3 | 0 | 0.0 |
| Dyspnea exertional | 3 | 0 | 0.0 |
| Ejection fraction decreased | 3 | 0 | 0.0 |
| Mitral valve incompetence | 3 | 2 | 66.7 |
| Sinus node dysfunction | 3 | 0 | 0.0 |
| Atrial tachycardia | 2 | 0 | 0.0 |
| Atrioventricular block complete | 2 | 0 | 0.0 |
| Bradycardia | 2 | 0 | 0.0 |
| Bundle branch block left | 2 | 0 | 0.0 |
| Cardiac arrest | 2 | 1 | 50.0 |
| Cardiac disorder | 2 | 1 | 50.0 |
| Cardio-respiratory arrest | 2 | 1 | 50.0 |
| Carotid artery stenosis | 2 | 0 | 0.0 |
| Circulatory collapse | 2 | 2 | 100.0 |
| Congestive cardiomyopathy | 2 | 1 | 50.0 |
| Cor pulmonale | 2 | 1 | 50.0 |
| Hypertension | 2 | 2 | 100.0 |
| Hypertensive heart disease | 2 | 2 | 100.0 |
| Multiple organ dysfunction syndrome | 2 | 0 | 0.0 |
| Pulmonary congestion | 2 | 1 | 50.0 |
| Right ventricular failure | 2 | 1 | 50.0 |
| Acute coronary syndrome | 1 | 1 | 100.0 |
| Acute respiratory distress syndrome | 1 | 1 | 100.0 |
| Anemia | 1 | 1 | 100.0 |
| Angina pectoris | 1 | 0 | 0.0 |
| Aortic valve stenosis | 1 | 1 | 100.0 |
| Arrhythmia supraventricular | 1 | 0 | 0.0 |
| Atrioventricular dissociation | 1 | 0 | 0.0 |
| Bradyarrhythmia | 1 | 0 | 0.0 |
| Bronchospasm | 1 | 0 | 0.0 |
| Bundle branch block right | 1 | 1 | 100.0 |
| Cardiac valve replacement complication | 1 | 1 | 100.0 |
| Cardiomegaly | 1 | 1 | 100.0 |
| Cardiorenal syndrome | 1 | 0 | 0.0 |
| Cor pulmonale chronic | 1 | 1 | 100.0 |
| Device malfunction | 1 | 1 | 100.0 |
| Endocarditis staphylococcal | 1 | 0 | 0.0 |
| Generalized edema | 1 | 0 | 0.0 |
| Hypoglycaemia | 1 | 0 | 0.0 |
| Hypoxia | 1 | 0 | 0.0 |
| Malnutrition | 1 | 1 | 100.0 |
| Medical device battery replacement | 1 | 0 | 0.0 |
| Non-cardiogenic pulmonary edema | 1 | 1 | 100.0 |
| Palpitations | 1 | 0 | 0.0 |
| Peripheral swelling | 1 | 0 | 0.0 |
| Pleural effusion | 1 | 1 | 100.0 |
| Postoperative respiratory failure | 1 | 0 | 0.0 |
| Pulmonary hypertension | 1 | 1 | 100.0 |
| Stress cardiomyopathy | 1 | 0 | 0.0 |
| Systolic dysfunction | 1 | 0 | 0.0 |
| Tachycardia | 1 | 0 | 0.0 |
| Transplant evaluation | 1 | 1 | 100.0 |
| Tricuspid valve incompetence | 1 | 1 | 100.0 |
| Troponin increased | 1 | 0 | 0.0 |
| Ventricular dyssynchrony | 1 | 1 | 100.0 |
| Ventricular fibrillation | 1 | 0 | 0.0 |
| Volvulus | 1 | 1 | 100.0 |
| Missing preferred term* | 124 | 58 | 46.8 |

Table is based on all events sent for adjudication. *Adjudicated event (e.g. from ECG, Lab) but missing adverse event information, MedDRA version 19.0. EAC, Event Adjudication Committee; ECG, electrocardiogram; MedDRA, Medical Dictionary for Regulatory Activities.

# Additional Table S2: hHF by system organ class

|  | **N** | **%** | **Events** | **Rate** |
| --- | --- | --- | --- | --- |
| **hHF events (SMQ definition)** | 372 | 4.9 | 550 | 3.64 |
| Cardiac disorders | 349 | 4.6 | 499 | 3.30 |
| General disorders and administration site conditions | 14 | 0.2 | 16 | 0.11 |
| Investigations | 1 | 0.0 | 1 | 0.01 |
| Respiratory, thoracic and mediastinal disorders | 29 | 0.4 | 33 | 0.22 |
| Vascular disorders | 1 | 0.0 | 1 | 0.01 |
|  | | | | |
| **hHF events (broad MedDRA definition)** | 618 | 8.1 | 948 | 6.27 |
| Cardiac disorders | 529 | 6.9 | 784 | 5.18 |
| Gastrointestinal disorders | 1 | 0.0 | 1 | 0.01 |
| General disorders and administration site conditions | 20 | 0.3 | 22 | 0.15 |
| Investigations | 1 | 0.0 | 1 | 0.01 |
| Metabolism and nutrition disorders | 6 | 0.1 | 10 | 0.07 |
| Nervous system disorders | 54 | 0.7 | 61 | 0.40 |
| Respiratory, thoracic and mediastinal disorders | 61 | 0.8 | 68 | 0.45 |
| Vascular disorders | 1 | 0.0 | 1 | 0.01 |

hHF, hospitalization for HF; MedDRA, Medical Dictionary for Regulatory Activities; N, number of patients; %, proportion of patients; Rate, events per 100 patient-years of exposure; SMQ, standardized Medical Dictionary for Regulatory Activities Query.
